# Southeast Asian diversity: first insights into the complex mtDNA structure of Laos

**DOI:** 10.1186/1471-2148-11-49

**Published:** 2011-02-18

**Authors:** Martin Bodner, Bettina Zimmermann, Alexander Röck, Anita Kloss-Brandstätter, David Horst, Basil Horst, Sourideth Sengchanh, Torpong Sanguansermsri, Jürgen Horst, Tanja Krämer, Peter M Schneider, Walther Parson

**Affiliations:** 1 Institute of Legal Medicine, Innsbruck Medical University, Müllerstrasse 44, Innsbruck, Austria; 2 Institute of Mathematics, University of Innsbruck, Technikerstrasse 13, Innsbruck, Austria; 3 Division of Genetic Epidemiology, Department of Medical Genetics, Molecular and Clinical Pharmacology, Innsbruck Medical University, Schöpfstrasse 41, Innsbruck, Austria; 4 Pathologisches Institut, Ludwig-Maximilians-Universität, Munich, Germany; 5 Department of Dermatology, Columbia University, New York, NY, USA; 6 National University of Laos, Vientiane, Laos; 7 Department of Pediatrics, Chiang Mai University, Chiang Mai, Thailand; 8 Institut für Humangenetik, Universität Münster, Münster, Germany; 9 Institute of Legal Medicine, University of Cologne, Melatengürtel 60-62, Cologne, Germany

## Abstract

**Background:**

Vast migrations and subsequent assimilation processes have shaped the genetic composition of Southeast Asia, an area of close contact between several major ethnic groups. To better characterize the genetic variation of this region, we analyzed the entire mtDNA control region of 214 unrelated donors from Laos according to highest forensic quality standards. To detail the phylogeny, we inspected selected SNPs from the mtDNA coding region. For *a posteriori *data quality control, quasi-median network constructions and autosomal STR typing were performed. In order to describe the mtDNA setup of Laos more thoroughly, the data were subjected to population genetic comparisons with 16 East Asian groups.

**Results:**

The Laos sample exhibited ample mtDNA diversity, reflecting the huge number of ethnic groups listed. We found several new, so far undescribed mtDNA lineages in this dataset and surrounding populations. The Laos population was characteristic in terms of haplotype composition and genetic structure, however, genetic comparisons with other Southeast Asian populations revealed limited, but significant genetic differentiation. Notable differences in the maternal relationship to the major indigenous Southeast Asian ethnolinguistic groups were detected.

**Conclusions:**

In this study, we portray the great mtDNA variety of Laos for the first time. Our findings will contribute to clarify the migration history of the region. They encourage setting up regional and subpopulation databases, especially for forensic applications. The Laotian sequences will be incorporated into the collaborative EMPOP mtDNA database http://www.empop.org upon publication and will be available as the first mtDNA reference data for this country.

## Background

Laos is the only landlocked country of Mainland Southeast Asia. Under Siamese control since the 18^th ^century, Laos became part of French Indochina in 1893 and gained full independence in 1954. The country was severely affected by the Vietnam War. In 1975, the Lao People's Democratic Republic was established. It has a population of almost seven million [1]. Southeast Asia is an area of close contact between several major ethno-linguistic groups: the Daic (Tai-Kadai), the Austro-Asiatic (including the Mon Khmer), the Sino-Tibetan (including Tibeto-Burmans and Han), the Hmong-Mien (Miao-Yao), the Austronesian and the Altaic. Migration and assimilation processes formed the genetic landscape of culturally separated ethnic groups living in the same geographic area [2-8]. Laos has an unusually high degree of human diversity [9]: more than 200 ethno-linguistic groups have been identified [1]. The population pattern is the result of vast movements in the last 2000 years, mainly from China to the southern lowlands. The highlands in the North and along the Annamite Cordillera are inhabited by minorities [2]. Laotian tribes have formerly been classified into three categories based on the ethno-linguistic family, the customary habitat and the type of agricultural production: Lao Lum ("lowlander") of the Tai-Kadai, Lao Thoeng/Teung/Kang ("mid- or uplander") of the Mon Khmer and Lao Sung/Song ("highlander") of the Sino-Tibetan and the Hmong-Mien. This classification ceased in 1981 due to promotion of unity of the nation and changes in habitat and agriculture [10]. The number of officially listed tribes varies: 68 prior to 1975, 47 in 1995 [11]. In 2000, an ethno-linguistic system of 49 groups was established [1]. In addition, the classification based on language and culture has some difficulties [2,8,10,12]. Genetic data from Laos are scarce. The great ethnic diversity specifies the requirements for a population sample. For forensic, population and phylogenetic purposes, it needs to cover the lineages that occur as comprehensive as possible. This is warranted by country-wide sampling. We examine the mtDNA composition of Laos for the first time, presenting a cross-sectional population sample of 214 individuals from throughout the country that was analyzed according to the highest forensic standards.

## Methods

### MtDNA samples and DNA extraction

**Figure 1:**
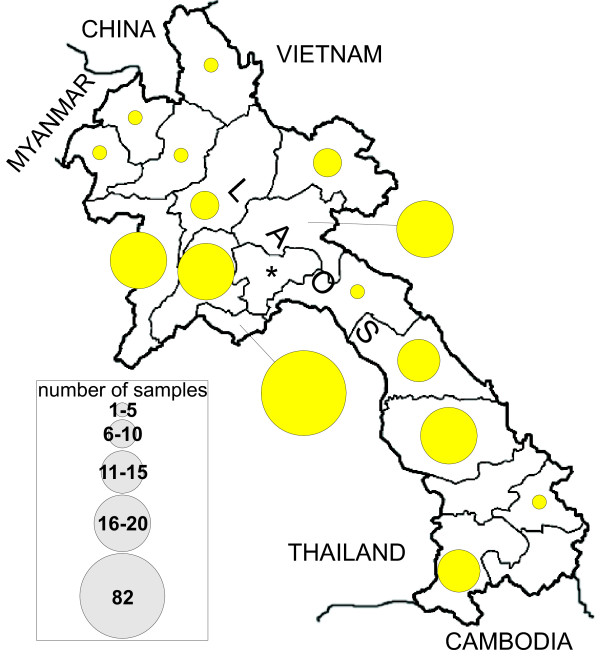
**Origin of the Laos samples**. Laos and its state and province borders are shown. The diameter of a circle is relative to the number of samples from the province it is assigned to. The asterisk indicates the former Xaisomboun (Saysomboune) military district dissolved in 2006.

220 blood samples were acquired from volunteers of both sexes from The Lao People's Democratic Republic. Total DNA was extracted from peripheral blood lymphocytes using automated standard protocols [13]. All donors gave their informed consent. Samples were fully anonymized. Ethical approval was obtained from the Lao People's Democratic Republic's National Ethics Committee for Health Research (No.89/NECHR). The donors' provinces of birth comprise 15 of the 17 provinces of Laos. Figure 1 shows the number of samples from each province. For a detailed list of donor provenience, see Additional file 1. No ethnic or linguistic affiliations are available. The dataset will support all genetic investigations regarding the total mtDNA variation found in Laos.

### MtDNA sequence analysis and haplogroup assignment

We generated full mtDNA control region (CR) sequence data of 220 samples by chain termination sequencing. All experiments were performed according to the EMPOP forensic standard procedures to ensure highest sequence quality and reliable base calling [14]. Contiguous CR sequences were assembled, phylogenetically aligned [15] and reported with respect to the revised Cambridge Reference Sequence [16] using Sequencher V4.8 (Gene Codes Corporation).

We performed partial coding region (codR) sequencing in order to refine the phylogeny of samples that could not be assigned to a haplogroup more specific than paragroups M*, N* or C*. For samples of paragroup M*, we examined up to four segments (nps 1820-2450, nps 4450-5580, nps 8670-9850, nps 11450-12820) comprising SNPs diagnostic for M haplogroups, haplogroups D, G, and subhaplogroups. Samples of paragroup C* were inspected for their haplogroup C4 and C7 status by sequencing nps 5820-6660. This fragment was also used to test for B4 subhaplogroups and D5b1, where relevant. To detect haplogroups of paragroup N*, we analyzed nps 4450-5860 and nps 6340-7770. Primer sequences and experimental conditions were applied as published before [17]. To scrutinize samples for haplogroups C1c, G3b, L3b or M13, CR sequencing primer reads were extended upstream to np 15900.

In order to endorse a reference haplogroup nomenclature, we used the comprehensive mtDNA "phylotree", release 8 [18], for assigning haplotypes to haplogroups according to their SNP pattern.

### Post sequencing data inspection

*A posteriori *data quality control was performed using the NETWORK software provided on the EMPOP website (http://www.empop.org, 2^nd ^release) [19,20]. This program facilitates the inspection of rare or unobserved substitutions and indels occurring in the dataset that could represent possible sequence errors [21]. The resulting HVS-I and HVS-II networks are shown in Additional file 2 and explained in Additional file 3.

Identical haplotypes (disregarding cytosine insertions after nps 16193, 309 and 573 and point heteroplasmies) were inspected for maternal relatedness by typing up to 17 autosomal STR loci using the AmpFℓSTR SGM Plus PCR Amplification Kit (Applied Biosystems) and the PowerPlex 16 System (Promega). Five samples were thereafter removed from the dataset, applying a cutoff likelihood ratio of 1000 [22]. One sample was excluded due to contamination.

### Population genetic analyses

In order to shed light on the genetic structure and population genetic history of Laos and Southeast Asia, the Laos sample was compared to surrounding populations. Both countrywide and ethnic subpopulation samples from recent literature were compiled [3-7,12,17,23-26]. All sequences were aligned and trimmed to a greatest common range, cytosine insertions after nps 16193 and 309 were disregarded. We conducted two separate analyses, one comprising a total of 1229 samples from this study, Northern Thailand [17], Vietnam [23], Hong Kong [24] and a mixed Han sample [7] with a greatest common range of nps 16024-16497 and 30-407 (see Table 1), another comprising Laos and 16 East Asian populations with a total of 5470 samples (nps 16024-16383) (see Table 2).

**Table 1:** Diversity measures of the Laos sample and four other East Asian populations (HVS-I and HVS-II)

Population statistics	Laos	Northern Thailand	Vietnam	Hong Kong	Mixed Han
Reference	This study	**Zimmermann 2009 **[17]	**Irwin 2008 **[23]	**Irwin 2009 **[24]	**Yao 2002 **[7]
Number of samples	214	190	187	376	262
Proportion of haplotypes	0.79	0.74	0.83	0.80	0.94
Proportion of unique haplotypes	0.64	0.58	0.73	0.69	0.89
Mean pairwise differences	10.932 +/- 4.990	10.790 +/- 4.932	10.620 +/- 4.859	10.652 +/- 4.862	10.964 +/- 5.000
RMP	0.008	0.011	0.009	0.005	0.004

Total N = 1229, nps 16024-16497 30-407

**Table 2:** Diversity measures of the Laos sample and 16 other East Asian populations (HVS-I)

Population statistics	Laos	Northern Thailand	Vietnam	Hong Kong	Austro-Asiatic	Daic
Reference	This study	**Zimmermann 2009 **[17]	**Irwin 2008 **[23]	**Irwin 2009 **[24]	**Li 2007 **[3]	**Li 2007 **[3]
Number of samples	214	190	187	376	124	772
Proportion of haplotypes	0.68	0.65	0.73	0.65	0.57	0.45
Proportion of unique haplotypes	0.53	0.50	0.59	0.51	0.38	0.31
Mean pairwise differences	7.686 +/- 3.597	7.498 +/- 3.518	7.353 +/- 3.455	7.291+/- 3.421	6.842 +/- 3.243	6.558 +/- 3.103
RMP	0.012	0.015	0.011	0.008	0.023	0.01

						

**Population statistics**	**Pinghua Han**	**Southern Indig. Minorities**	**Tibet**	**Southern Tibeto-Burman**	**Guangdong Han**	**Mixed Han**
**Reference**	**Gan 2008 **[12]	**Gan 2008 **[12]	**Wen 2004 **[4]	**Wen 2004 **[4]	**Chen 2008 **[25]	**Yao 2002 **[7]

Number of samples	197	273	91	405	106	262
Proportion of haplotypes	0.71	0.58	0.69	0.69	0.89	0.87
Proportion of unique haplotypes	0.54	0.42	0.47	0.53	0.80	0.79
Mean pairwise differences	7.918 +/- 3.697	7.536 +/- 3.529	6.317 +/- 3.024	7.759 +/- 3.621	7.178 +/- 3.392	7.620 +/- 3.566
RMP	0.012	0.018	0.02	0.007	0.013	0.006

						

**Population statistics**	**Hmong**	**Mien**	**Northern Han**	**Southern Han**	**Island South East Asians**	
**Reference**	**Wen 2005 **[5]	**Wen 2005 **[5]	**Wen 2004 **[6]	**Wen 2004 **[6]	**Hill 2007 **[26]	

Number of samples	167	370	238	473	1025	
Proportion of haplotypes	0.63	0.51	0.82	0.74	0.40	
Proportion of unique haplotypes	0.50	0.36	0.74	0.63	0.27	
Mean pairwise differences	6.269 +/- 2.991	6.574 +/- 3.113	6.860 +/- 3.240	7.407 +/- 3.469	6.716 +/- 3.170	
RMP	0.022	0.016	0.009	0.005	0.012	

Total N = 5470, nps 16024-16383

The random match probability for each population was calculated as sum of squared haplotype frequencies (disregarding cytosine insertions after nps 16193, 309 and 573). We performed intra- and interpopulation comparisons: the number of mean pairwise differences within and between populations, molecular diversity indices, and an analysis of molecular variance (AMOVA) were calculated using Arlequin (version 3.5.1.2) [27]. Also the corrected numbers of MPD between populations were determined, i.e. the MPD between two populations minus the mean number of MPD contained within these two populations. To give additional perspective on the genetic relation of the populations and for a visualization of the AMOVA results, we performed a correspondence analysis based on pairwise F*_ST _*values using PASW Statistics 18 (SPSS Inc.).

## Results and Discussion

### Haplogroup composition, unique and most common haplotypes of the Laos sample

The 214 Laotian samples classified into 64 distinct haplogroups [18]. 45% of the samples were assigned to a terminal twig of the "phylotree". 171 CR haplotypes, of which 141 were unique (66% of the samples) were detected (disregarding indels around nps 16193, 309 and 573). The most prevalent haplogroups were B5a (12%), F1a1a (7.5%), C7 and M7b1 (6% each). The most frequent CR haplotypes (five occurrences each) were a B5a (73-210-263-315.1C-523del-524del-16140-16183C-16189-16266A-16519) and a F1a1 haplotype (73-249del-263-315.1C-523del-524del-16129-16162-16172-16304-16399-16519).

Macrohaplogroup N (including haplogroups A, B, F, N and R) comprised 57% of the samples in 37 haplogroups. 26% of the samples were assigned to haplogroup B, almost equally to B4 and B5. 26 out of the 27 haplogroup B5 samples were found to be haplogroup B5a. 22% of the samples belonged to haplogroup F, of which 79% belonged to F1a and its subhaplogroups. Macrohaplogroup M (including haplogroups C, D, G and M) comprised 43% in 27 haplogroups. 32% of the samples belonged to haplogroup M, distributed among ten subhaplogroups. 25% of the M samples, however, remained M*. No maternal west Eurasian or African admixture was detected.

**Figure 2:**
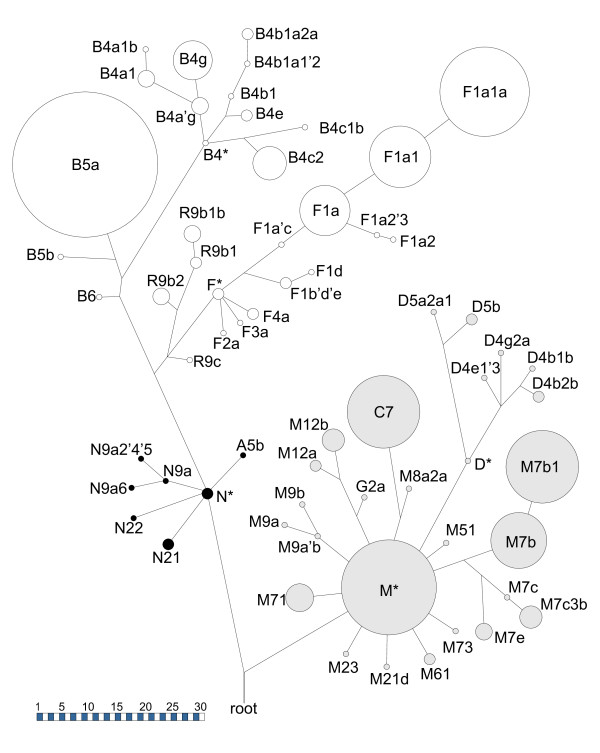
**Schematic phylogenetic tree of all mitochondrial haplogroups found in the Laos sample (N = 214)**. Haplogroups according to [18], release 8. The circles' sizes correspond to the haplogroup frequencies. The diameter bar indicates the number of samples. Prehaplogroups are denoted as haplogroups, stem lengths are of no information content. The tree is rooted in the most recent common ancestor. *Gray circles*: descendants of haplogroup M; *black circles*: descendants of haplogroup N; *empty circles*: descendants of haplogroup R.

Figure 2 depicts a phylogenetic tree of all haplogroups and their absolute frequencies. A haplogroup and frequency list is given in Table 3. The analyzed range, haplotype and haplogroup of every sample are available from Additional file 4.

**Table 3:** MtDNA haplogroup frequencies within 214 samples from Laos

Haplogroup	N	Frequency (%)
A5b	1	0.5
B4*	1	0.5
B4a'g*	3	1.4
B4a1*	3	1.4
B4a1b	1	0.5
B4b1*	1	0.5
B4b1a1'2*	1	0.5
B4b1a2a	2	0.9
B4c1b	1	0.5
B4c2	6	2.8
B4e	2	0.9
B4g	7	3.3
B5a	26	12.1
B5b	1	0.5
B6	1	0.5
C7	13	6.1
D*	1	0.5
D4b1b	1	0.5
D4b2b	2	0.9
D4e1'3	1	0.5
D4g2a	1	0.5
preD5a2a1	1	0.5
D5b	2	0.9
F*	2	0.9
F1a'c*	1	0.5
F1a*	9	4.2
F1a1*	11	5.1
F1a1a	16	7.5
F1a2	1	0.5
F1a2'3*	1	0.5
F1b'd'e*	2	0.9
F1d	1	0.5
F2a	1	0.5
F3a	1	0.5
F4a	2	0.9
G2a	1	0.5
M*	17	7.9
M7b*	10	4.7
M7b1	13	6.1
M7c*	1	0.5
M7c3b	4	1.9
M7e	3	1.4
M8a2a	1	0.5
M9a'b*	1	0.5
M9a	1	0.5
M9b	1	0.5
M12a	2	0.9
M12b	4	1.9
M21d	1	0.5
preM23	1	0.5
M51	1	0.5
preM61	2	0.9
M71	5	2.3
M73	1	0.5
N*	2	0.9
N9a*	1	0.5
N9a2'4'5	1	0.5
N9a6	1	0.5
preN21	2	0.9
N22	1	0.5
R9b1*	2	0.9
R9b1b	3	1.4
R9b2	3	1.4
R9c	1	0.5

Haplogroups according to [18], release 8. Frequencies are rounded to one decimal place.

### Diversity indices of the Laos sample

The power of discrimination by CR was calculated 99.26%, the random match probability 0.74%. The number of mean pairwise differences for full CR was 13.14 ± 5.94. This high number is due to the double origin of East Asian mtDNA lineages in both macrohaplogroup M and N.

### Point heteroplasmic positions in the Laos sample

Point heteroplasmies at a single CR position were observed in eight samples. Two of these (16093Y and 152Y) were the most common point heteroplasmic positions observed in a dataset of 5015 global samples [28]. One position (16261Y) was found three times, two positions a single time (16289R and 16468Y), three heteroplasmic positions (16086Y, 16179Y, 16271Y) were absent in the 5015 samples. A search in the 10970 sequences (including most of the 5015) of the EMPOP database (http://www.empop.org, 2^nd ^release) yielded three hits for 16086Y, the latter two positions remained unobserved. The proportion of CR profiles revealing a heteroplasmic position was 3.7%, in agreement with observations in blood samples from other populations (1-9.5%) [28].

### New insights into the Southeast Asian mtDNA phylogeny

After CR sequencing, a number of Laos samples displayed new SNP motifs within haplogroups B and D that were found recurrent in other Central, East and Southeast Asian populations (Thailand, Vietnam, Hong Kong, Uzbekistan, Japan and the Miao) [17,23,24,29-31]. These findings (see Table 4 and Additional file 5) indicate that the phylogeny of the particular lineages is not yet fully resolved.

**Table 4:** Novel haplogroup B and D mtDNA CR SNP motifs found recurrent in Laos and other Asian populations

Haplogroup	Additional CR motif	Found in Laos and
B4	183-310-374-16274-16289-16301	[23]
B4g	61A-62-16181C-16213	[29]
B6	234	[23]
D	247	[17]
D4g2a	@195	[17,30]

Haplogroups according to [18], release 8.

The partial codR sequencing had a great impact on the phylogenetic resolution of the dataset. It helped to confirm and exclude haplogroups without or with recurrent CR motifs. In addition, new codR SNP patterns were detected. The assays applied therefore constitute valuable time-, cost- and DNA-saving alternatives to whole mitochondrial genome sequencing for phylogenetic purposes.

**Figure 3:**
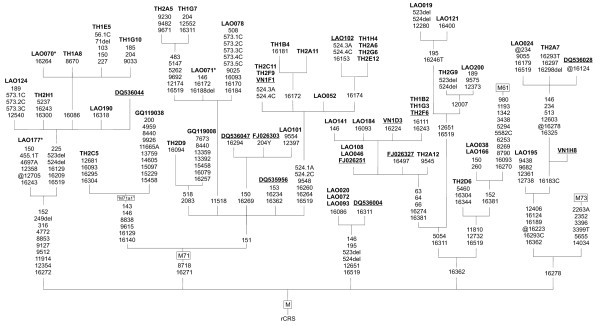
**Phylogenetic tree of new haplogroup M lineages detected in this study and surrounding populations**. All SNPs detected are indicated (disregarding cytosine insertions after nps 16193, 309 and 315). Haplogroups M61 and M73 are depicted to show possible phylogenetic relations. Haplogroups according to [18], release 8. *Sequenced range: *full mtDNA CR and optional codR covering either (a) all SNPs, (b) most SNPs - for asterisked samples or (c) no SNPs - for underlined samples. For details, see Additional file 4. *References: *this study, [17,23,24,32], P.M. Schneider, personal communication.

The paragroup M* codR sequencing assay was performed on 41 samples. 14 samples were confirmed to belong to a described M haplogroup, haplogroups D or G. New, recurrent SNP motifs with matching haplotypes from Thailand, Vietnam, Hong Kong and the Philippines [[17,23,24,32] and P.M. Schneider, personal communication] were revealed by all but two of the remaining M* samples. Figure 3 shows the novel M lineages found. To remain with the established nomenclature [18], we refrained from assigning new haplogroup names. For some haplotypes, codR SNPs that were not analyzed were inferred from the known information. This strategy is widely applied when CR is only sequenced partly [4,5,7], and in genetic epidemiology ("imputing").

The recently described haplogroup M71 was diverse in the Laos sample. Several new sublineages (including the postulated "M71a1" [32]) could be identified in 23 individuals from Laos, Thailand, Vietnam, China and the Philippines. Three haplogroup M samples from Laos and Thailand revealed the SNP pattern 11810-12732-16362 indicating haplogroup M61 [33], while other presumably diagnostic SNPs were not present. These findings indicate that the Southeast Asian mtDNA phylogeny is far from being resolved and needs more sequence information for full clarification (see Figure 3).

We inspected the recurrent codR SNPs for their effect on amino acid level using the "MitoWheel" v1.2 http://www.mitowheel.org. Interestingly, two out of the 11 base substitutions caused amino acid substitutions (valine-isoleucine and vice versa), while nine were silent mutations. SNP variants persistent in the face of purifying selection are expected to be silent, adaptive or neutral (by affecting weakly conserved amino acids or causing substitutions by similar amino acids, as encountered here) [34]. This finding could be another indication of a true phylogenetic meaning of the new basal haplogroup M codR SNP patterns that we found in multiple samples from several populations.

### Haplogroup composition of Laos compared to surrounding populations

The Laos sample showed mtDNA diversity characteristic of Southeast Asian populations. The composition of haplogroups was in agreement with other populations from this region [3-7,12,17,23-25,35], with haplogroups B4a, B5a, M7b1, F1a and R9 being the most frequent southern aboriginal lineages. The ample haplogroup pattern may reflect the rich history of migrations of ethnic groups in today's Laos. Aside from a few haplogroups with high sample numbers, a plethora was present at very low frequencies. The five most prevalent haplogroups together comprised 37% of the samples (see Table 3).

In Laos, haplogroups B4a and R9 were less frequent than expected from the literature. This is partly explained by "technical" reasons: samples assigned to haplogroup "R9a" by HVS-I-motifs [4,12] actually belong to F3a (R9a is discontinued). Nevertheless, there was only one F3a sample in the Laos dataset. Earlier publications considered B4g haplotypes as B4a due to their common root [3,5,7,12,23,35]. B4g, not B4a is the most prevalent B4 haplogroup in Laos.

Little Northern contribution was detected. The presence of haplogroups described as Northern (East) Asian [4,6,7,25,36], i.e. A, Z, Y, C, M8a, M9, G2, D and N9, was low in the Laos dataset. Haplogroups G, M8 and A were present as singletons, Y and Z were absent. Haplogroup C, in contrast, showed an unexpectedly high frequency of 6%, but very limited diversity: all haplotypes belonged to haplogroup C7. Interestingly, 12 of the 13 samples derive from Northern provinces (see Additional file 1). Together with the singular presence of haplogroup M8, this is indicative of a founder effect - possibly the immigration of a small group of females carrying the C7 haplogroup from its Northern pool.

### Genetic comparisons with four East Asian populations (HVS-I and HVS-II)

We compared the genetic composition of the current sample to that of populations from Thailand [17], Vietnam [23], Hong Kong [24] and mixed Han from China and Taiwan [7] with the greatest common range of nps 16024-16497 and 30-407. The total number of samples was 1229 (see Table 1).

The numbers of intrapopulation MPD were highly similar. The highest intrapopulation diversity was found in the mixed Han sample (10.96) followed by Laos (10.93); the lowest in Vietnam (10.62). The intrapopulation RMP ranged between 0.004 (Han) and 0.011 (Thailand) indicating that the populations were highly differentiated. Laos was intermediate with a value of 0.008 (see Table 1). Interpopulation MPD ranged from 10.68 (Vietnam-Hong Kong) to 11.18 (Laos-Han), the corrected values from 0 (Laos-Vietnam) to 0.23 (Laos-Han). The low and similar values indicate similar genetic structures of the populations. The corrected and uncorrected results corresponded, as population pairs ranked low, medium or high in both calculations (see Additional file 6).

All populations shared haplotypes to varying extents. 47 haplotypes (28%) of the Laos sample were found in the other populations. Thailand was at the top (16% shared haplotypes) followed by Vietnam (12%) and Hong Kong (8%). The mixed Han sample only shared 3% of its haplotypes with Laos and its most frequent shared haplotype was only a singleton in the Laos sample (see Additional file 7).

AMOVA was used to test for significant variation in the mtDNA genetic structure among the populations. Almost all genetic variation observed is attributable to differences within populations (99.16%). Variance among populations only accounts for 0.84%. The AMOVA results were statistically significant (see Additional file 6).

The low and similar pairwise F*_ST _*values possibly reflect the common origin of the populations and little evolutionary time since their differentiation. However, the small genetic variance detected was significant for almost all comparisons. No significant difference in genetic structure was found between the Laos and the Vietnam population sample, which may indicate extensive gene flow by migration between the two countries (see Additional file 6).

### Genetic comparisons with 16 East Asian populations (HVS-I)

To shed more light on the genetic structure of the Laos sample, we performed analogous analyses including a greater number of East Asian populations [3-7,12,17,23-26] with a shorter segment (nps 16024-16383). The total number of samples was 5470 from 17 populations (see Table 2).

The numbers of intrapopulation MPD ranged from 6.27 to 7.92. The highest intrapopulation diversity was found in the Pinghua Han [12] (confirming their genetically heterogeneous background), the lowest in the Hmong [5]. Laos was third highest (7.69). The intrapopulation RMP ranged from 0.005 (Southern Han [6]) to 0.023 (Austro-Asiatic [3]) indicating highly differentiated populations. Laos was intermediate at 0.012 (see Table 2).

The interpopulation MPD spanned from 6.47 (Hmong [5]-Daic [3]) to 7.90 (Southern Tibeto-Burmans [4]-Pinghua Han [12]), the corrected values from 0 (even arithmetical values below zero) for several comparisons to 0.74 (Laos-Tibet [4]). The range of values was greater than for the analyses using a longer segment (see previous section), revealing a greater population differentiation. This was expected given the broader geographic origin of populations included. Tibet, being a population at far geographic distance, had the highest numbers of MPD with all of the samples but the other Northern sample (Northern Han [6]), an effect that was only visible after correction (see Additional file 8).

The order of the populations in the MPD and RMP analyses was different when only HVS-I was analyzed (see previous section), which demonstrates the impact of larger reading frames. It can be supposed that the results of analyses with more information included are closer to truth.

All populations shared haplotypes with the Laos sample. 46% of the Laos haplotypes were found in other populations. This high percentage is due to the short mtDNA segment and the high number of populations involved. The geographically closer populations, i.e. Thailand [17], Hong Kong [24], the Southern Indigenous Minorities [12], Pinghua Han [12], Guangdong Han [25], Vietnam [23], Hmong and Mien [5] and the Austro-Asiatic [3] tended to share higher proportions (13-30%) than the Southern Tibeto-Burmans and Tibet [4], Daic [3], the mixed Han samples [6,7] and the sample from Island Southeast Asia [26] (3-9%) (see Additional file 7).

AMOVA again showed that the observed genetic variation was mainly attributable to differences within populations (98.09%). Variance among populations accounted for 1.91% (see Additional file 8). The AMOVA results were statistically significant.

Mainly low, but also some intermediate F*_ST _*values resulted from the pairwise comparisons, indicating limited genetic differentiation between the populations (as in the previous section). All intermediate F*_ST _*values were yielded in comparisons including the Tibet [4] or the Northern Han [6] sample, and comparisons with these two population samples resulted in the highest pairwise F*_ST _*values for most other groups. The outlier position of the two Northern samples detected in several genetic comparisons supports the concept of isolation by geographic distance with subsequent differentiation.

Most differences in genetic structure were highly significant. Only the mixed Han sample [7] and the Pinghua Han [12] with multi-ethnic origin showed no significant difference in several comparisons, along with the Laos sample when compared to the Vietnam sample [23], as shown in the previous section (see Additional file 8).

**Figure 4:**
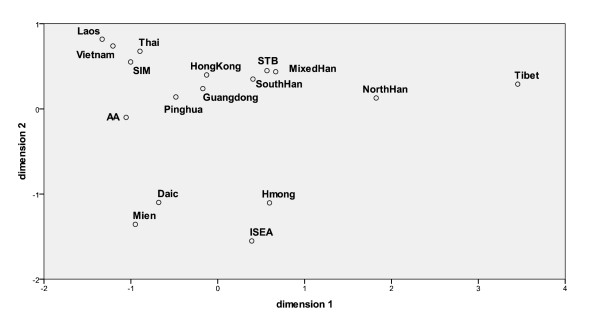
**Multidimensional scaling analysis of genetic distances between 17 population samples from East Asia**. Computed from F*_ST _*indices based on nps 16024-16383 (see Additional file 8). Stress value: 0.085. *Abbreviations: *AA - Austro-Asiatic, ISEA - Island Southeast Asians, SIM - Southern Indigenous Minorities, STB - Southern Tibeto-Burmans. *References: *[3-7,12,17,23-26].

An MDS plot for visualization of the AMOVA results is depicted in Figure 4. The positioning of the samples did not change when nonsignificant F*_ST _*values (see Additional file 8) were excluded. In this correspondence analysis, the population sample from Laos clustered with the samples from Vietnam [23] and Thailand [17] and the Southern Indigenous Minorities [12]. This might be explained by the fact that these four samples do not represent ethnic entities, but are a similar combination of several groups. The partly shared political history of the three neighboring countries could have facilitated migrations.

The other populations appeared more distant from the Laos sample: the Austro-Asiatic [3] and the cluster of the Guangdong Han [25], Pinghua Han [12] and the sample from Hong Kong [24] were closer, the cluster of Southern Han [6], Mixed Han [7] and Southern Tibeto-Burmans [4] at an intermediate distance, while the remaining groups, i.e. the Hmong and Mien [5], the Daic [3], the Northern samples (Northern Han [6], Tibet [4]) as well as the Island Southeast Asians [26] localized further away.

Obviously, the Han population samples did not cluster in the correspondence analysis. Although assigned to the same nationality, they are distant from each other genetically. The mixed Han [7] localized between the Northern and Southern Han [6] - as expected near their sister Sino-Tibetan branch, the Southern Tibeto-Burman sample [4] - while the Pinghua [12] and the Guangdong [25] Han samples with minority background and the sample from Hong Kong [24] clustered between the other Southeast Asian populations rather than with the other Han samples. This reflects the process of assimilation of indigenous populations along with the Han expansion [12,25] and the particular genetic composition of Hong Kong [24] possibly caused by a history distinct from the rest of today's China. Our findings further point out the importance of subdatasets even for large ethnic groups, as reported before [6,7]. It was also meaningful to separate the Hmong and Mien population samples [5], that are usually combined based on linguistics, as they differ genetically (see Figure 4).

### Understanding the genetic history of Laos

The rapid initial colonization of Southeast Asia at some time after 60 kYBP along the "Southern Coastal Route" was followed by an expansion *in situ *(while other groups moved on), a dispersal into the continent and onto the islands, and the subsequent differentiation of ethnic groups with common origin but diverging lineages (most of which arose during the last stadial of the Würm glacial, 30-10 kYBP, probably in different refugia) [3,7,26,37,38]. We confirmed this in our analyses: the novel basal M haplogroups found in high diversity in the Laos sample and surrounding populations support the fast migration and *in situ *differentiation model (see Figure 3). Despite of little evolutionary time, the linguistically separated groups also clearly differed genetically (see Figure 4).

The original genetic structure of MSEA with distinct areas inhabited by the main ethnic groups - the Daic in the coastal areas of the Southeast, the Austro-Asiatic in Western and central MSEA, the Sino-Tibetan (Tibeto-Burmans and Han) in the North and the Hmong-Mien in today's central China - has been blurred by massive migrations [3,7]. In the past 2600 years, the Tibeto-Burmans moved from the Northwest and the Himalayan area to the South, absorbing indigenous lineages [4]. In the past 2000 years, several waves of Han expansions to the West and South caused massive displacements of indigenous minorities [3,4,6,7]. The Daic were forced southward by the expanding Han in a fanning spread and are now mainly found west of Hong Kong. In today's Laos, they formed small city states in the North from the late 11^th ^century AD and later moved to the central and Southern plains, thereby repelling the Austro-Asiatic population to the highlands or assimilating them. Today, the Daic "lowlander" living in the plains and along the rivers constitute the major proportion (60-65%) of the Laos population. They are dominant in language, culture, media and politics [1-3,6,10,39]. The Hmong-Mien are the newest arrival: they were continuously forced to the Southwestern areas already settled by Austro-Asiatic and Southern Tibeto-Burmans. They immigrated to the hilly North of Laos only in the past several hundred years and today exhibit a pattern of refuge ("highlander"), rather than a positive expansion [2,5,8].

Traces of these migrations might be visible in the extant mtDNA composition of Laos. To shed more light on the population genetic history, it was meaningful to compare the cross-sectional Laos sample to those of the ethnic groups that together constitute the population of this country.

An interesting picture was revealed (see Additional Files 6, 7 and 8, Figure 4): the ethnic population with the highest similarity to the Laos sample in terms of shared haplotypes, MPD, pairwise F*_ST _*values and localization in the MDS plot were the Austro-Asiatic [3]. This was unexpected, as the "midlander" only constitute 20-25% of the Laotian population, being the major group only in some regions [10,39]. Given the fact that the Daic sample [3] localized at much further distance from the Laos sample in the correspondence analysis (see Figure 4), had a higher corrected MPD value and less shared haplotypes, our findings indicate a great proportion of assimilated Austro-Asiatic lineages in the Daic-dominated Laos.

The ethnic samples second closest to the Laos sample in the MDS plot were the three "highlander" Southern Sino-Tibetan groups (Southern Tibeto-Burmans [4], Southern [6] and Mixed Han [7]), while other "highlander", the Hmong and Mien [5], appeared far from the Laos sample. This might reflect expanding Sino-Tibetan tribes immigrating from the Northwest of MSEA, and is in agreement with the reported migration routes (see above). Both in the shared haplotypes analysis (see Additional File 7) and the MDS plot (see Figure 4), there was only small evidence for admixture by Northern Sino-Tibetan groups (Tibet [4] and North Han [6]), suggesting that these migrations were minor concerning the area of today's Laos or blurred by the admixture by indigenous females to immigrating Northern groups (as reported for the Southern Han [6]). Specific, but limited Northern traces were found in the Laos sample, however (haplogroups D, M8 and C7, see above).

## Conclusion

The cross-sectional Laotian sample presented here is highly suitable for genetic purposes regarding the countrywide mtDNA variation. Subpopulation and regional databases for detailed population genetic investigations and reliable forensic frequency estimates are desirable and need more extensive sampling. New phylogenetic lineages were detected: this sample will significantly contribute to further clarification of the Southeast Asian mtDNA phylogeny and the development of region specific filters for NETWORK constructions [19,20]. The limited codR sequence analysis considerably increased phylogenetic resolution, suggesting that complete mitochondrial genome sequencing and analyzing a greater number of samples and populations will help to identify additional new lineages, yielding a more realistic picture of human mtDNA diversity, dispersal history and a higher power of discrimination for forensic purposes.

This first Laos dataset reveals a highly diverse population in terms of mtDNA composition, possibly reflecting the contribution of several major ethnolinguistic groups and a complex migration history. The Laotian population sample showed to be highly differentiated and lies well amid other Southeast Asian populations in terms of haplogroup structure, diversity indices and sharing of haplotypes. Nevertheless, its genetic structure was significantly different from 15 East Asian groups included in the comparisons. Our sample gives strong indication for a mixed Southeast Asian aboriginal origin of the extant Laos population along with limited Northern East Asian contributions, with highest similarity to Austro-Asiatic and Southern Sino-Tibetan populations. This finding was unexpected, as the Daic are culturally dominant in Laos, but is in agreement with the ethnic divisions of Southeast Asia before the Han expansions, where the Austro-Asiatic lived in this central area of today's Laos [3]. However, this mtDNA study reflecting the maternal history of Laos is only one piece of the puzzle. Y-chromosomal and autosomal markers will allow further, and possibly contrary, insights into the complex migration and population history of Laos and Southeast Asia [40,41].

The haplotypes presented in this study will be available on the EMPOP database http://www.empop.org [EMPOP:EMP00083] and on GenBank [GenBank:HM852213-HM852426] upon publication. This publication follows the recommendations of the International Society of Forensic Genetics on the use of mtDNA in forensic analyses.

## List of abbreviations used

AMOVA: analysis of molecular variance; CR: control region (of the mtDNA); codR: coding region (of the mtDNA); d.f.: degrees of freedom; HVS: hypervariable segment; kYBP: thousand years before present; MDS: multidimensional scaling; MPD: mean pairwise differences; MSEA: Mainland Southeast Asia; mtDNA: mitochondrial DNA; np: nucleotide pair; RMP: random match probability; SNP: single nucleotide polymorphism; STR: short tandem repeat.

## Authors' contributions

MB designed and conducted experiments, retrieved data, performed calculations, interpreted results and conceived and wrote the manuscript. BZ conducted experiments, interpreted results and contributed to the manuscript. AR retrieved data, performed calculations, interpreted results and contributed to writing and editing the manuscript. AK helped in organizing DNA samples and contributed to the manuscript. DH, BH, SS, TS and JH helped in conceiving the study, organized DNA samples, performed DNA extractions and contributed to the manuscript. TK performed laboratory work and contributed to the manuscript. PS provided data and contributed to the manuscript. WP conceived and coordinated the entire project, supervised experiments, interpreted and validated results and contributed to writing and editing the manuscript. All authors approved the final version of the manuscript.

## Supplementary Material

Additional file 1 **Donor provenience list**. The Laos province of birth is given for every donor. Click here for file

Additional file 2 **Quasi-median network portraits from 214 mtDNA control regions from Laos**. Network torsos of HVS-I (nps 16024-16569; part A) and HVS-II (nps 1-576; part B). Condensed and filtered haplotypes are represented by the nodes, their haplogroup and number is indicated. Prehaplogroups are denoted as haplogroups. A plus sign indicates that haplotypes of several haplogroups have been condensed in that node. Transitions are marked in green, transversions in red. Click here for file

Additional file 3 **Interpretation of quasi-median networks from 214 mtDNA control regions from Laos**. In this file, the networks in Additional file 2 are explained. Click here for file

Additional file 4 **MtDNA polymorphisms of the 214 samples from Laos**. The analyzed range, haplotype (as full list of differences to the revised Cambridge Reference Sequence [16]) and haplogroup of every sample are given. Haplogroups according to [18], release 8. Click here for file

Additional file 5 **Refined phylogeny of haplogroup B4e**. This modified CR phylogeny is indicated by five samples from Laos, Vietnam [23], Hong Kong [24] and Japan [31]. The tree is rooted in haplogroup B4. Haplogroups according to [18], release 8. Click here for file

Additional file 6 **AMOVA results for five East Asian populations (analyzed range: nps 16024-16497 30-407)**. Part A: Design and results of AMOVA. Part B: Population average pairwise differences. Part C: F*_ST _*comparison among populations. *References*: this study, [7,17,23,24] Click here for file

Additional file 7 **Shared haplotypes within the Laos population sample**. Only one example haplotype is indicated per shared haplotype. Part A: comparison with four East Asian populations (nps 16024-16497 30-407). Part B: comparison with 16 East Asian populations (nps 16024-16383). *References*: this study, [3-7,12,17,23-26]. Click here for file

Additional file 8 **AMOVA results for 17 East Asian populations (analyzed range: nps 16024-16383)**. Part A: Design and results of AMOVA. Part B: Population average pairwise differences. Part C: F*_ST _*comparison among populations. *References*: this study, [3-7,12,17,23-26]. Click here for file
