# Supplementary figures and images for: Southeast Asian diversity: first insights into the complex mtDNA structure of Laos
Source: BMC Evol Biol. 2011 Feb 18;11:49. doi: 10.1186/1471-2148-11-49 (PMC3050724; doi:10.1186/1471-2148-11-49)

A

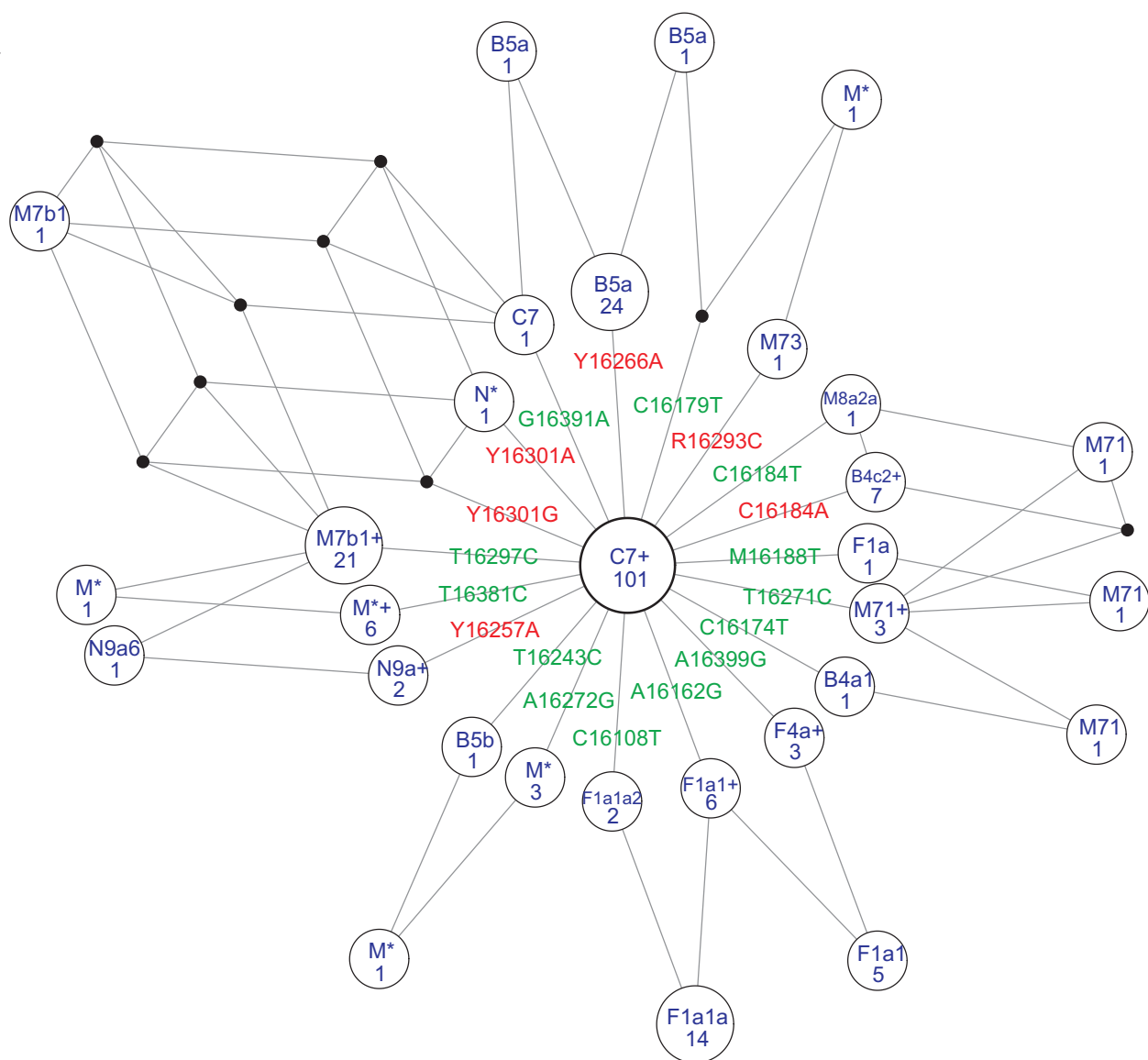

B

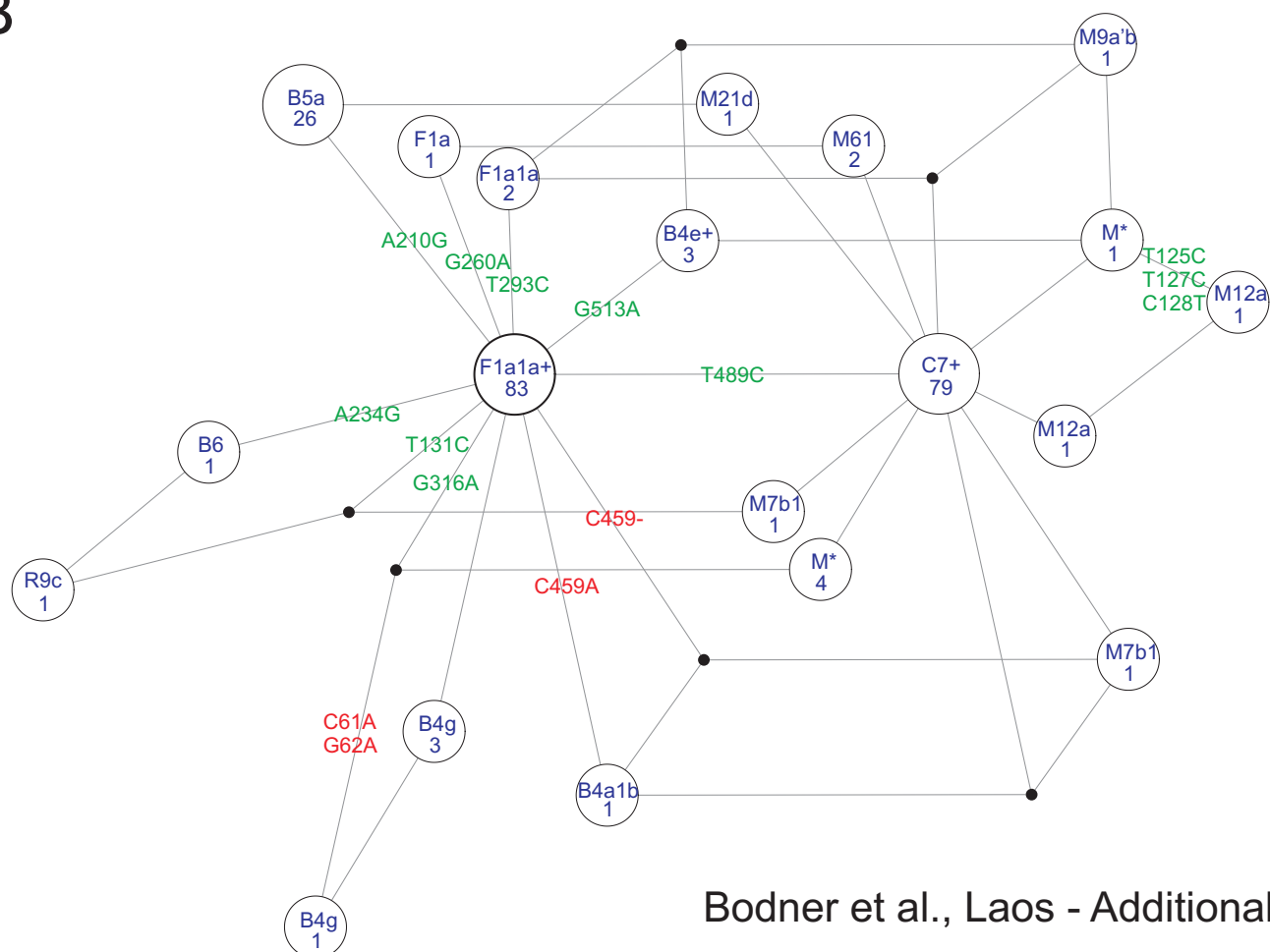

Supplement: Additional file 2 — Quasi-median network portraits from 214 mtDNA control regions from Laos. Network torsos of HVS-I (nps 16024-16569; part A) and HVS-II (nps 1-576; part B). Condensed and filtered haplotypes are represented by the nodes, their haplogroup and number is indicated. Prehaplogroups are denoted as haplogroups. A plus sign indicates that haplotypes of several haplogroups have been condensed in that node. Transitions are marked in green, transversions in red. [file 1471-2148-11-49-S2.PDF]

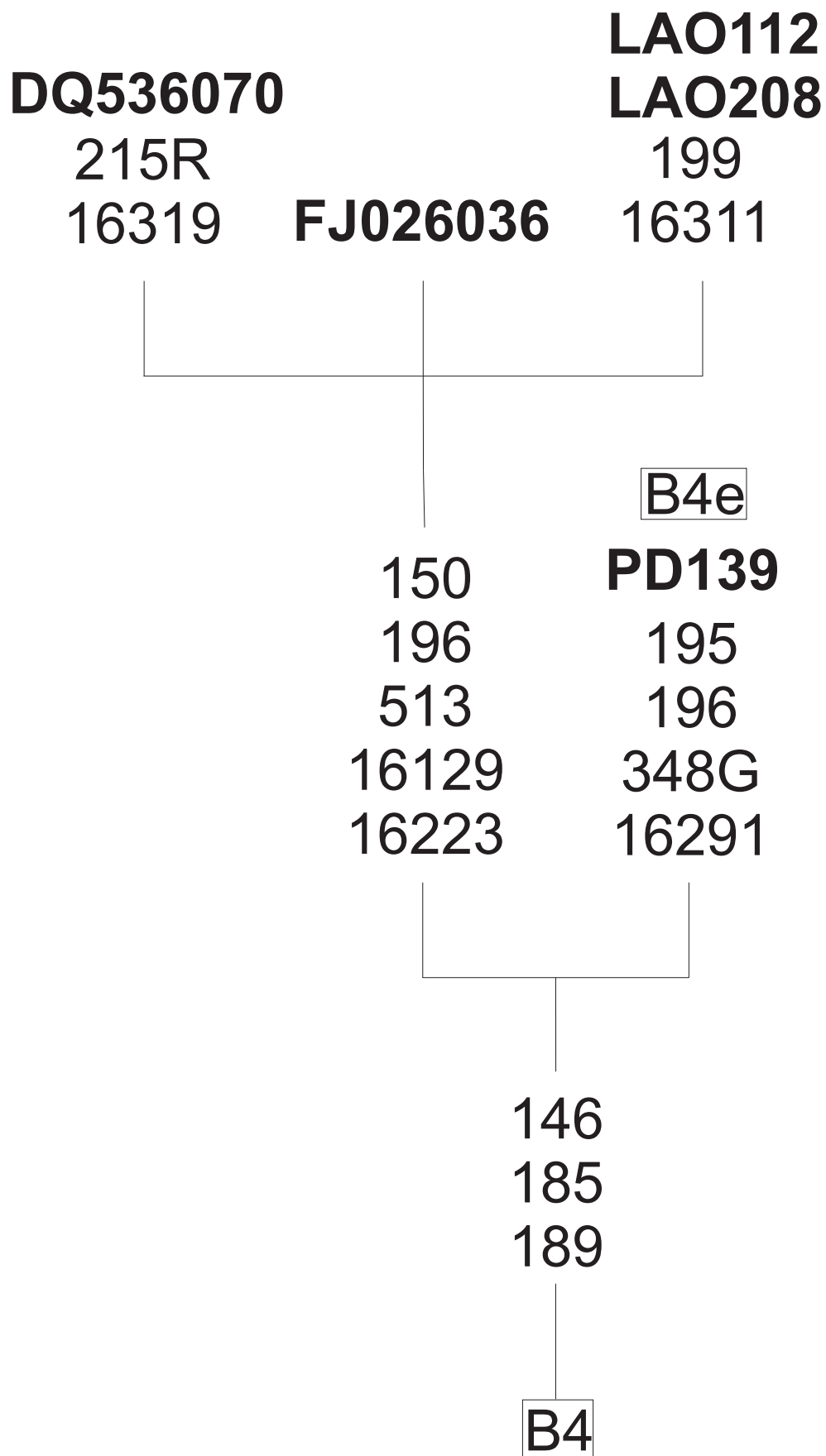

Supplement: Additional file 5 — Refined phylogeny of haplogroup B4e. This modified CR phylogeny is indicated by five samples from Laos, Vietnam [23], Hong Kong [24] and Japan [31]. The tree is rooted in haplogroup B4. Haplogroups according to [18], release 8. [file 1471-2148-11-49-S5.PDF]
