# Supplementary material for: Southeast Asian diversity: first insights into the complex mtDNA structure of Laos
Source: BMC Evol Biol. 2011 Feb 18;11:49. doi: 10.1186/1471-2148-11-49 (PMC3050724; doi:10.1186/1471-2148-11-49)
Supplement: Additional file 3 — Interpretation of quasi-median networks from 214 mtDNA control regions from Laos. In this file, the networks in Additional file 2 are explained. [file 1471-2148-11-49-S3.PDF]

Bodner et al., Laos: Additional file 3

Quasi-median network analysis: Interpretation of results

A posteriori data quality control was performed using the NETWORK software provided on the EMPOP website (http://www.empop.org) [19,20]. This program facilitates the inspection of rare or unobserved substitutions and indels occurring in the dataset that could represent possible sequence errors. Network analyses were performed separately for HVS-I (nps 16024-16569) and HVS-II (nps 1-576). The resulting network torsos are shown in Additional file 2. Following the guidelines of [42], the sequences were condensed to the weighty variation applying the EMPOP speedy filter for hypervariable polymorphisms [20] before computations. Heteroplasmic positions were specified to the phylogenetic or major variant.

The HVS-I network torso of the Laos dataset exhibits the expected star-like structure (see Additional file 2, part A). Complex structures are caused (a) by ternary and quaternary sites where not all occurring mutations are filtered, e.g. variants 16184A and 16184T in haplotypes of haplogroups B4c2, M8a2a and M71 and variants 16301A and 16301G in haplotypes of paragroup N* and haplogroup M7b1 and (b) by variants occurring in multiple samples with different haplogroup background, as 16174T in B4a1 and M71 or 16391A in B5a and C7. The HVS-II network of this (and any) East Asian data set is divided into two distinct sections that comprise the descendants of macrohaplogroup M (right section) and N (left section), respectively, partitioned by the transition at np 489. Asian specific SNPs occuring independently in both macrohaplogroups thus cause triangular prisms and cuboid structures (see Additional file 2, part B), as the existing filters are adapted for Western Eurasian sequences. The Laos population data will contribute to a regional filter for reliable NETWORK inspection.
